# Inhibition of KDM4C/c‐Myc/LDHA signalling axis suppresses prostate cancer metastasis via interference of glycolytic metabolism

**DOI:** 10.1002/ctm2.764

**Published:** 2022-03-28

**Authors:** Ching‐Yu Lin, Bi‐Juan Wang, Yu‐Ke Fu, Chieh Huo, Ya‐Pei Wang, Bo‐Chih Chen, Wei‐Yi Liu, Jen‐Chih Tseng, Shih Shen Jiang, Zong‐Lin Sie, Kelvin K Tsai, Chiou‐Hwa Yuh, Wen‐Ching Wang, Hsing‐Jien Kung, Chih‐Pin Chuu

**Affiliations:** ^1^ Institute of Cellular and System Medicine National Health Research Institutes Miaoli Taiwan; ^2^ PhD Program for Cancer Molecular Biology and Drug Discovery Taipei Medical University Taipei Taiwan; ^3^ Immunology Research Center National Health Research Institutes Miaoli Taiwan; ^4^ National Institute of Cancer Research National Health Research Institutes Miaoli Taiwan; ^5^ College of Medicine Graduate Institute of Clinical Medicine Taipei Medical University Taipei City Taiwan; ^6^ Division of Gastroenterology Department of Internal Medicine Laboratory of Advanced Molecular Therapeutics Wan Fang Hospital Taipei Medical University Taipei, Taiwan; ^7^ Department of Life Science Institute of Molecular and Cellular Biology, National Tsing‐Hua University Hsinchu Taiwan; ^8^ Graduate Program for Aging China Medical University Taichung Taiwan; ^9^ Biotechnology Center National Chung Hsing University Taichung Taiwan

Dear Editor, we discovered that knockout of KDM4C can effectively suppress prostate cancer (PCa) cells’ migration and invasion. We identified that targeting KDM4C/c‐Myc/LDHA signalling can be an effective prevention for metastatic PCa. Epigenetics change is an important feature in cancer metabolism reprograming and metabolism rewiring can enhance cancer progression. Histone lysine demethylase 4C (KDM4C), which can remove the dimethyl/trimethyl group from H3K9 or H3K36, is an androgen receptor (AR) co‐regulator. KDM4C is elevated in castration‐resistant prostate cancer (CRPC)^1^ and elevation of KDM4C stimulates the proliferation of PCa cells.^2^ However, how KDM4C regulates PCa metastasis or cancer metabolism is unclear. We examined the KDM4C gene level in online gene datasets Gene Expression Omnibus (GEO) profile GDS 2547 (HG‐U95C) (Figure 1A), The Cancer Genome Atlas (TCGA) Prostate Adenocarcinoma (PRAD) (Supplemental Figure S1), Chandran PCa dataset (Figure 1B), Grasso PCa dataset (Figure 1C) and LaTulippe PCa dataset (Figure 1D). Expression level of KDM4C gene was increased in metastatic prostate cancer (PCa). To study how KDM4C promotes PCa metastasis, CRISPR/Cas9 single‐guide RNA (sgRNA) was constructed in C4‐2B PCa cells (Supplemental Figure S2), which impaired KDM4C's demethylation ability (Supplemental Figure S3). Knockout of KDM4C suppressed C4‐2B cells’ ability to migrate and to invade (Figure 1E). Knockdown of KDM4C with siRNA also repressed the migration and invasion of LNCaP cells (Figure 1F). Treatment with SD70, a potent and selective inhibitor for KDM4C, repressed the migration and invasion of both C4‐2B and LNCaP (Figure 1G–J) cells. KDM4C knockout decreased metastatic distance of C4‐2B PCa tumours in zebrafish xenotransplantation model (Figure 1K–M). Oppositely, overexpression of KDM4C promoted the migration of DU‐145 cells (Supplemental Figure S4).

**FIGURE 1:**
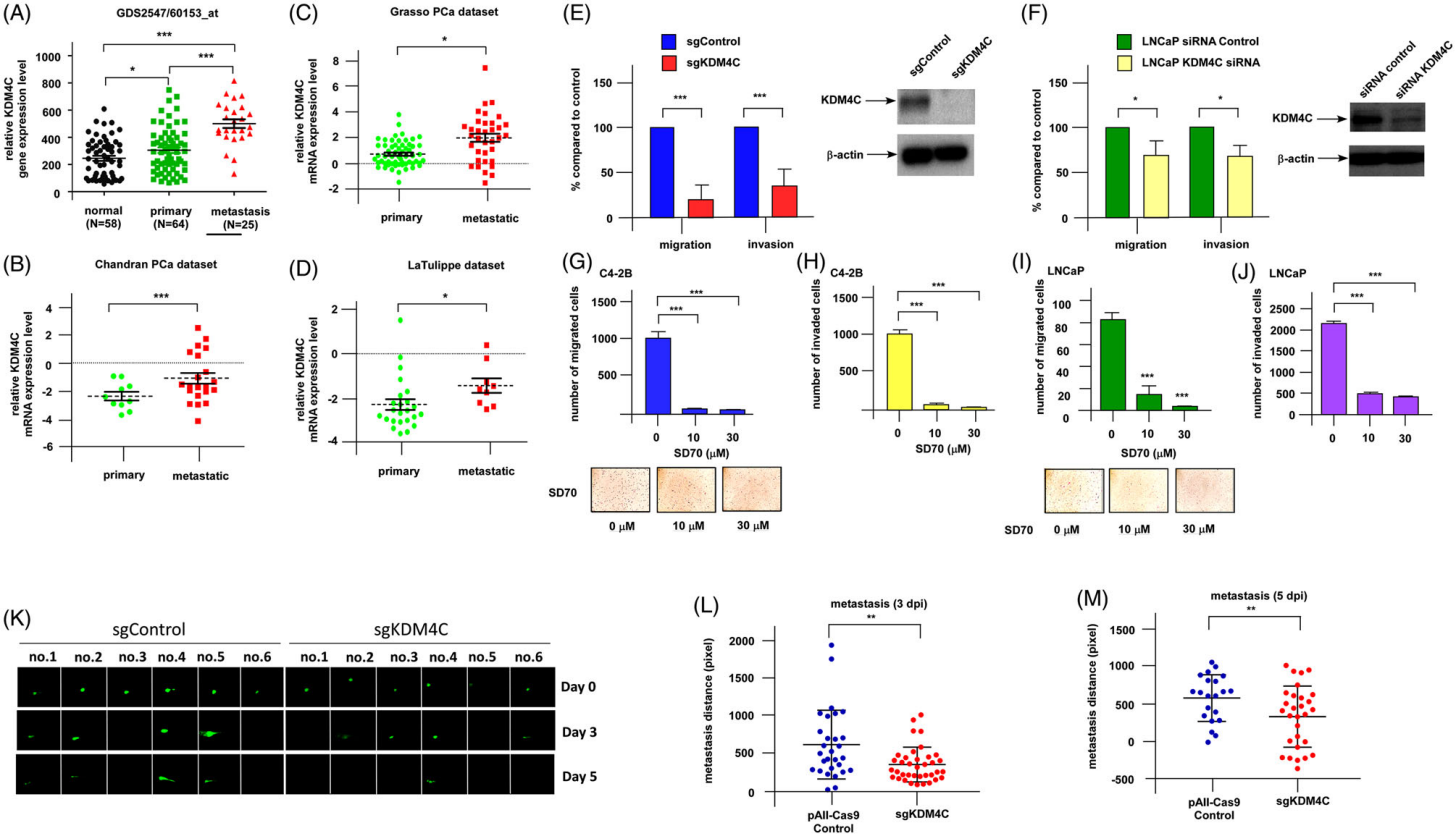
KDM4C expression is higher in metastatic prostate tumours as compared to primary prostate tumours and knockout of KDM4C suppresses migration and invasion of PCa cells both in vitro and in vivo. (A) GEO profiling of KDM4C gene expression level in adjacent normal prostate tissues, primary prostate tumours and metastasis prostate tumours extracted from GEO profile dataset GDS 2547 (HG‐U95C) (reporter: GPL93, 60153_at; gene ID: 23081). (B) Chandran Prostate dataset included 10 primary site prostate tumours and 21 metastatic prostate carcinomas. Expression of KDM4C gene was detected by CodeLink UniSet Human 20K I Bioarray (reporter: GE84522).^8^ (C) Grasso Prostate dataset included 59 primary site prostate tumours and 35 metastatic prostate carcinomas. Expression of KDM4C gene was detected by Agilent Human Genome 44K (reporter: A_32_P38313).^9^ (D) LaTulippe Prostate dataset included 23 primary site prostate tumours and 9 metastatic prostate carcinomas. Expression of KDM4C gene was detected by Human Genome U95A‐Av2 Array (reporter: 34980_at).^10^ (E) Migration and invasion of control C4‐2B (sgControl) cells and C4‐2B cells with CRISPR KDM4C knockout (sgKDM4C) was determined by transwell assay. (F) Migration and invasion of control LNCaP cells and LNCaP cells with KDM4C siRNA knockdown was determined by transwell assay. Western blotting confirmed the knockdown of KDM4C while β‐actin was used as loading control. Migration (G) and invasion (H) of C4‐2B cells being treated with 0, 10, 30 μM SD70 (KDM4C inhibitor) was examined by transwell assay. Migration (I) and invasion (J) of LNCaP cells being treated with 0, 10, 30 μM SD70 was examined by transwell assay. (K) Zebrafish xenotransplantation metastatic distance assay was used to compare the metastatic ability of sgControl cells (n = 48) and sgKDM4C cells (*n* = 48). Cells were injected into zebrafish yolk sac and xenotransplantation metastatic distance assay was measured three days post injection or five days post injection. The distribution and migration of sgControl and sgKDM4C cells in fish on the day of injection, 3rd and 5th day were shown in photography images. The xenotransplantation metastatic distance assay was measured three days post injection (dpi) (L) or five days post injection (M). Asterisks *, ** and *** represent statistically significantly different between the two groups being compared with *p* < .05, *p* < .01 and *p* < .001, respectively

To explore how KDM4C regulates PCa metastasis, gene microarray (Clariom S HT06, human gene microarray) was used to compare the gene profile in control C4‐2B (sgControl) cells versus KDM4C knockout C4‐2B cells (sgKDM4C). There were 441 genes being upregulated and 345 genes being downregulated (GEO number: GSE178727). Gene Set Enrichment Analysis (GSEA) analysis indicated that MYC target is the pathway most affected by KDM4C knockout (Figure 2A and B. Supplemental Figures S5 and S6). The suppression of *MYC* target genes by KDM4C knockout was confirmed by qRT‐PCR (Supplemental Figure S7). Micro‐Western Array was applied to analyse the changes of proteins involved in c‐Myc signalling, epithelial‐mesenchymal transition (EMT), glycolysis, TCA and oxidative phosphorylation (OXPHOS), cancer stemness and other metabolic proteins under influence of KDM4C knockout in C4‐2B cells (Supplemental Figure S8) or KDM4C knockdown in LNCaP cells (Supplemental Figure S9). Silencing of KDM4C repressed c‐Myc as well as proteins regulating EMT and metabolism (Figure 2C–F). Gene expression level of KDM4C and *MYC* were positively correlated in Chandran Prostate dataset (*r* = 0.595) and Grasso Prostate dataset (*r* = 0.477) (Supplemental Figure 10A and B). Chromatin‐immunoprecipitation (Ch‐IP) assay indicated that KDM4C directly interacted with *MYC* promoter region as knockout of KDM4C decreased 54% of the *MYC* promoter activity (Figure 3A) and repressed the *MYC* gene expression level (Figure 3B). We further constructed the upstream region of nucleotide 1–1160 of the *MYC* promoter region for luciferase reporter gene assay. Overexpression of KDM4C increased the transcription of *MYC* gene for approximately 23% (Figure 3C), while treatment with SD70 inhibitor decreased *MYC* gene transcription (Figure 3D). Ectopic expression of c‐Myc rescued the cell migration in sgKDM4C C4‐2B cells to level comparable to the sgControl (Figure 3E and F).

**FIGURE 2:**
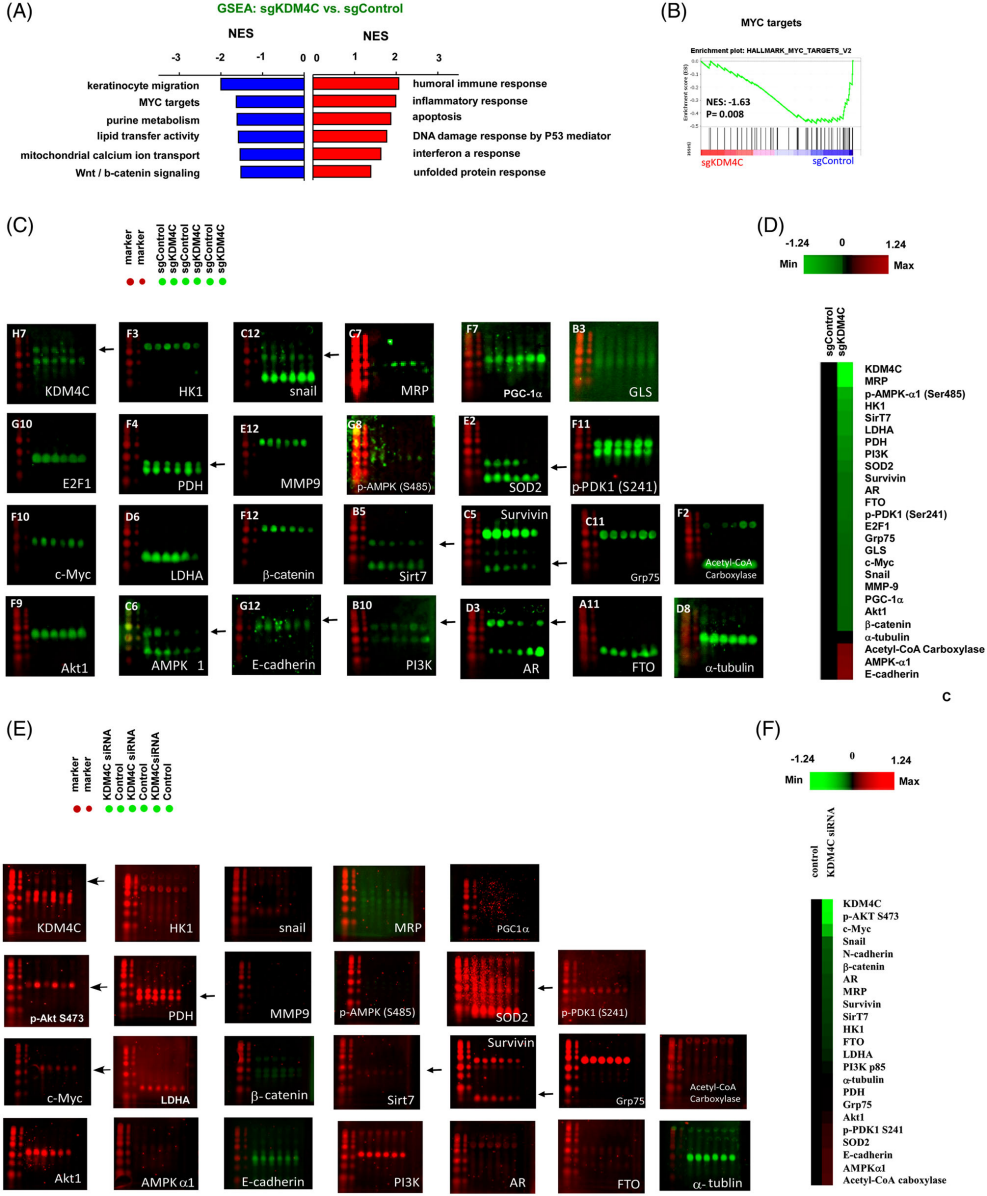
Gene Set Enrichment Analysis (GSEA) analysis and Micro‐Western Array (MWA) analysis revealed that knockout of KDM4C affected c‐Myc, c‐Myc target genes and proteins involved in metabolic pathways. (A) Top six pathways of upregulation and downregulation normalized enrichment score (NES) gene sets which fulfill the statistical criteria in sgKDM4C vs. sgControl cells were demonstrated. (B) Enrichment score plots of *MYC* target genes set was presented. Normalized enrichment score (NES) absolute value > 1, *p* < .05 and false discovery rate (FDR) < 0.25 was considered as reliable enrichment. (C) MWA was performed to analyse the difference in expression level of 96 antibodies targeting epithelial‐mesenchymal transition (EMT) marker proteins, proteins involved in glycolysis, tricarboxylic acid (TCA) cycle and oxidative phosphorylation (OXPHOS), and protein regulating cancer stemness in control C4‐2B cells and sgKDM4C C4‐2B. The results of MWA for proteins with significant changes were shown. (D) Heatmap was shown to demonstrate the proteins expression level with 1.2 fold increase or decrease in CRISPR knockout (sgKDM4C) cells as compared to control (sgControl) C4‐2B cells. Protein level was demonstrated using log_2_ value. (E) MWA was performed to analyse the difference in expression level of 96 antibodies targeting EMT marker proteins, proteins involved in glycolysis, TCA cycle and OXPHOS and protein regulating cancer stemness in control LNCaP cells and LNCaP with KDM4C siRNA knockdown. The results of MWA for proteins with significant changes were shown. (F) Heatmap was shown to demonstrate the proteins expression level with 1.2 fold increase or decrease in KDM4C knockdown LNCaP cells as compared to control LNCaP cells. Protein level was demonstrated using log_2_ value

**FIGURE 3:**
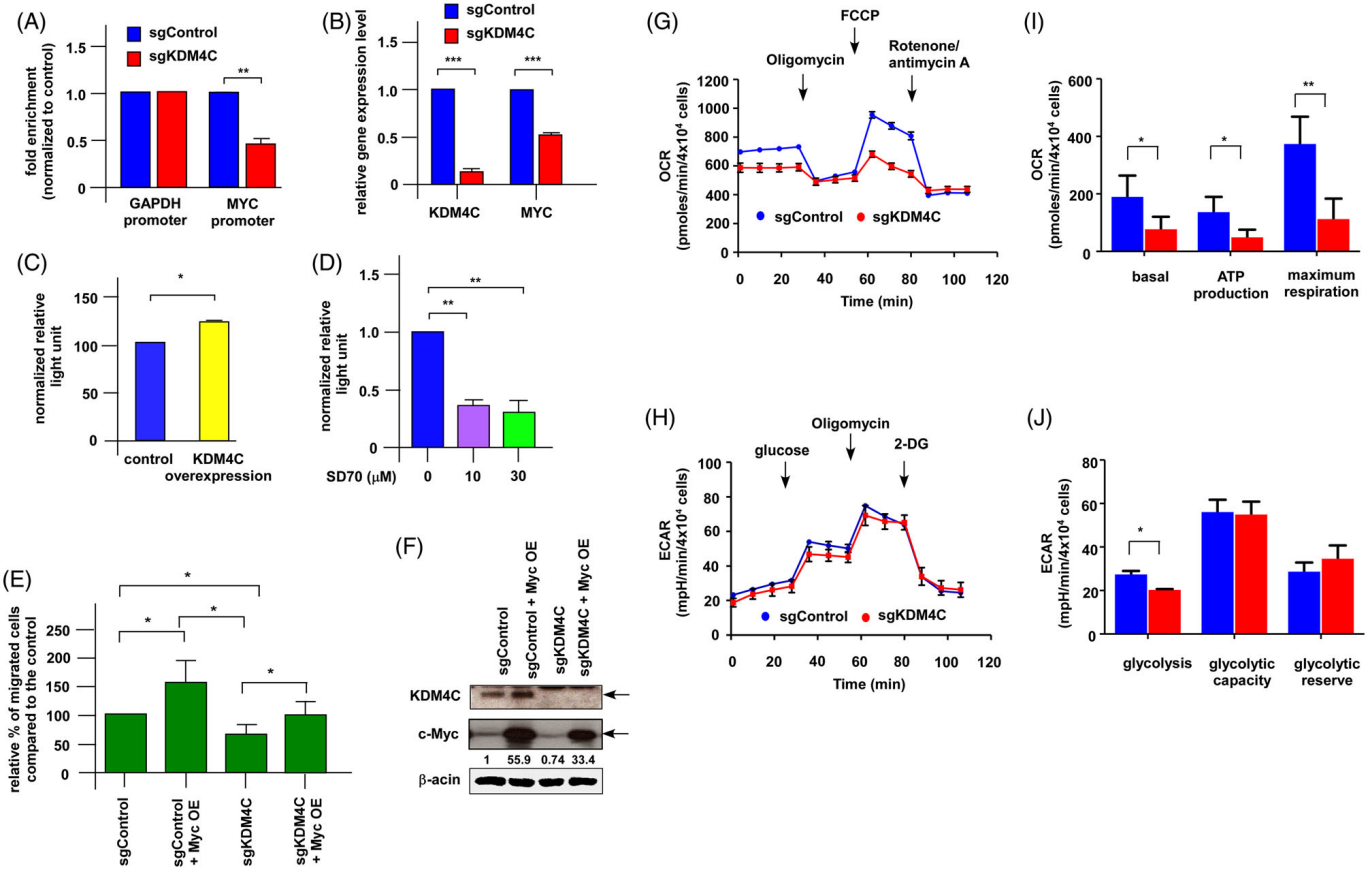
KDM4C directly binds to promoter of *MYC* and regulates PCa migration via c‐Myc while knockout of KDM4C affected mitochondrial function. (A) Chromatin immunoprecipitation analysed of binding between KDM4C protein and *MYC* promoter or control GAPDH promoter in sgControl and sgKDM4C C4‐2B cells. (B) Gene expression level of KDM4C and *MYC* in control C4‐2B and sgKDM4C C4‐2B cells was examined by qRT‐PCR. (C) Activity of *MYC* promoter in control C4‐2B cells and C4‐2B cells overexpressing KDM4C was determined by reporter gene assay. (D) Activity of *MYC* promoter in C4‐2B cells being treated with 0, 10, 30 μM of SD70 was determined by reporter gene assay. (E) Migration of sgControl or sgKDM4C C4‐2B cells with or without c‐Myc overexpression was measured by transwell assay. (F) Western blotting confirmed the knockout of KDM4C and overexpression of c‐Myc in sgControl cells and sgKDM4C cells. Function of mitochondria metabolism in sgControl C4‐2B cells (blue color) and sgKDM4C C4‐2B cells (red color) was examined by Seahorse platform for oxygen consumption rate (OCR) (G) and extracellular acidification rate (ECAR) (H). (I) Basal respiration, ATP production and maximum respiration of the mitochondria in sgControl cells and sgKDM4C cells was calculated from OCR trace in (G). (J) The glycolysis, glycolytic capacity and glycolytic reserve in sgControl cells and sgKDM4C cells was calculated from ECAR trace in (H). Asterisks *, ** and *** represent statistically significantly different between the two groups being compared with *p* < .05, *p* < .01 and *p* < .001, respectively

Seahorse metabolism platform was performed to examine effects of KDM4C knockout on mitochondrial functions. KDM4C knockout suppressed OCR (oxygen consumption rate) and ECAR (extracellular acidification rate) of mitochondria (Figure 3G and H) as well as suppressed production of ATP, glycolysis, basal respiration and maximum respiration rate (Figure 3I and J) in PCa cells. These observations suggested that sgKDM4C cells were under stress and becoming quiescent. Indeed, the proliferation rate of sgKDM4C cells was lower than that of sgControl cells (Supplemental Figure S11). As activated c‐Myc is known to induce the expression of several glycolytic enzymes,^3^ we examined the consequences of KDM4C knockout on expression of metabolic genes and proteins . Knockout of KDM4C suppressed gene and protein expression of GAPDH, hexokinase 2 (HK2), lactate dehydrogenase (LDH), LDHA, LDHB, phosphofructokinase‐1 (PFK1), Transaldolase 1 (TALDO1), Aconitase 1 (ACO1), Malate Dehydrogenase 1 (MDH1), pyruvate dehydrogenase E1 component subunit α (PDHA), PDH, pyruvate kinase M2 (PKM2), GLS and phosphogluconate dehydrogenase (PGD) but increased genes expression of γ‐glutamyltransferase 1 (GGT1) and glucose‐6‐phosphate dehydrogenase (G6PD) (Figure 4A and B). These genes and proteins are involved in regulation of glucose transporter and glycolysis, TCA cycle, lipid synthesis, metabolism of glutamine and pentose phosphate pathway. Knockout of KDM4C also reduced protein and gene expression of epithelial‐mesenchymal transition (EMT) regulatory proteins (Figure 4B and C). LDHA is a direct target of c‐Myc.^4^ LDHA converts pyruvate, which is derived from glycolysis of glucose, to lactate with simultaneous inter‐conversion of NADH and NAD^+^.^5^ Activation of c‐Myc increases the transport of glucose, its catabolism to trioses and pyruvate and production of lactate via induction of LDHA.^5^ Overexpression of LDHA in sgKDM4C C4‐2B cells restored the cell migration to level comparable to sgControl (Figure 4D and E), while knockdown of LDHA repressed migration of sgKDM4C cells (Figure 4F–G). Knockout of KDM4C reduced the secretion of lactate (Figure 4H) and increased the accumulation of pyruvate (Figure 4I) in sgKDM4C cells. Increase of secreted lactate will acidify the microenvironment nearby and assist cancer metastasis. Inevitably, lactate can be an inducer of cancer invasion and metastasis.^6^ PKM2 is an important enzyme in glycolysis. PKM2 has been reported to promote metastasis of PCa cells.^7^ Liquid chromatography‐mass spectrometry was applied to examine the difference in profile of metabolites inside the sgControl vs. sgKDM4C C4‐2B cells (Supplemental Figure S12). LC MS/MS and qRT‐PCR (Figure 4A) demonstrated that knockout of KDM4C decreased the enzymes and metabolites involved in glycolytic metabolism (Figure 4J). Our observations revealed that inhibition of KDM4C/c‐Myc/LDHA signalling suppresses PCa metastasis via suppression of glycolytic metabolism in PCa cells and targeting KDM4C/c‐Myc/LDHA signalling can be a potential therapy for advanced PCa.

**FIGURE 4:**
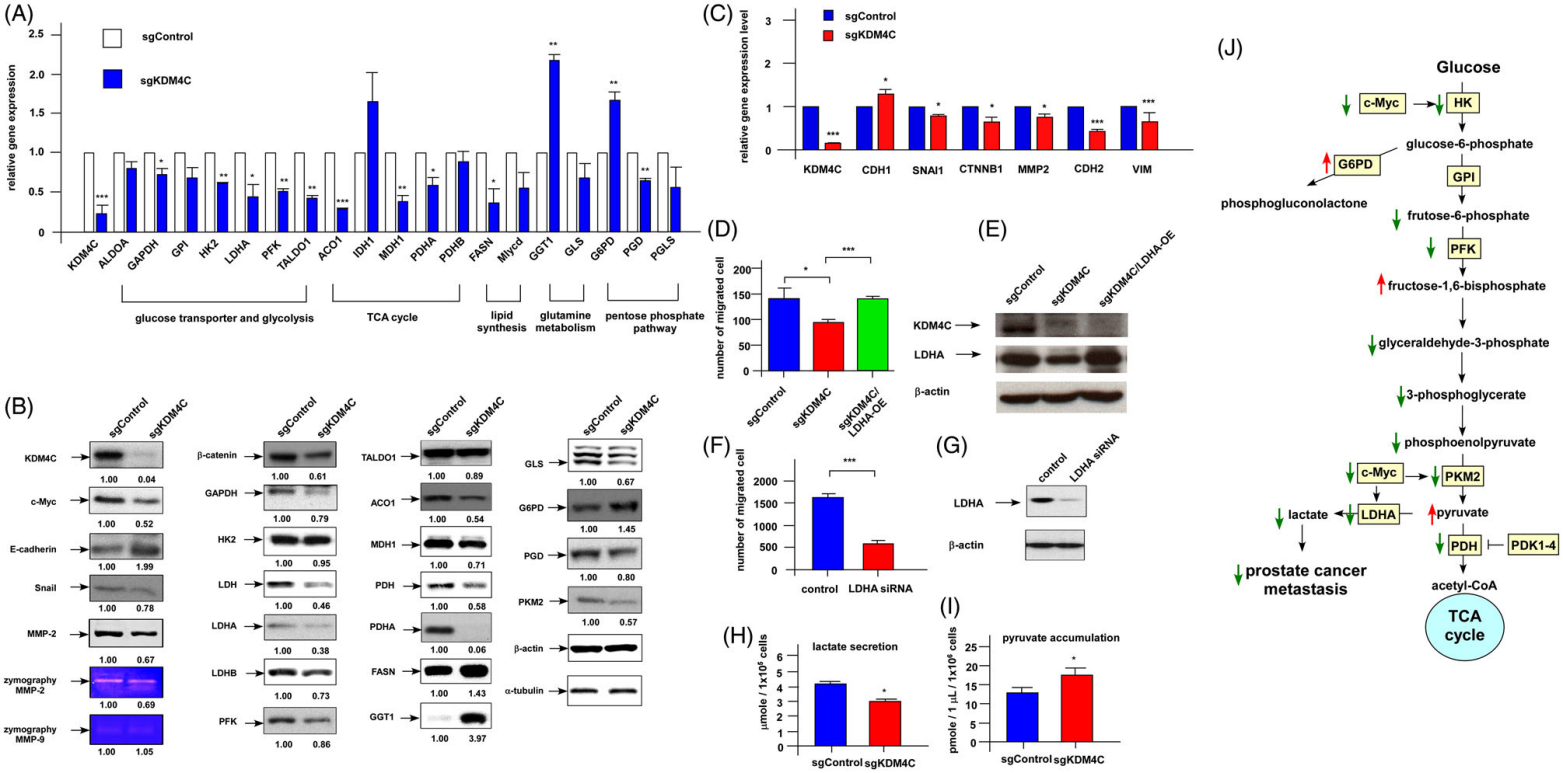
Knockout of KDM4C affects expression of metabolic genes and proteins as well as suppresses migration of PCa cells via repression of LDHA and decrease of lactate secretion. (A) Expression levels of metabolic genes, including genes regulating glucose transporter and glycolysis (*ALDOA*, *GAPDH*, *GPI*, *HK2*, *LDHA*, *PFK*, *TALDO1*), gene regulating TCA cycle (*ACO1*, *IDH1*, *MDH1*, *PDHA*, *PDHB*), gene regulating lipid synthesis (FASN, Mlycd), genes regulating glutamine metabolism (*GGT1*, *GLS*) and genes regulating pentose phosphate pathway (*G6PD*, *PGD*, *PGLS*) in sgControl and sgKDM4C cells was examined by qRT‐PCR. (B) Western blot analysis of expression levels of important proteins involved in metabolism and EMT in sgControl cells and sgKDM4C cells, including KDM4C, c‐Myc, GAPDH, HK2, PFK, TALDO1, ACO1, MDH1, FASN, GGT1, PKM2, PDH, G6PD, LDH, LDHA, LDHB, GLS, PGD, β‐catenin, E‐cadherin, Snail, MMP‐2. Zymography of MMP‐2 and MMP‐9 was examined. Expression level of β‐actin and α‐tubulin was used as loading control. (C) Gene expression of *KDM4C*, *CDH1*, *SNAl1*, *CTNNB1*, *MMP2*, *CDH2* and *VIM* was examined in sgControl vs. sgKDM4C cells with qRT‐PCR. (D) Migration of sgControl cells, sgKDM4C cells and sgKDM4C cells with LDHA overexpression was examined by transwell assay. (E) Overexpression of LDHA was confirmed by Western blotting assay. (F) Migration of C4‐2B cells with or without LDHA siRNA knockdown was examined by transwell assay. (G) Knockdown of LDHA was confirmed by Western blotting assay. Secretion of lactate (H) and accumulation of pyruvate (I) was measured in sgControl cells and sgKDM4C ells. (J) Effects of KDM4C knockout on metabolites and proteins in glycolytic metabolism pathway were shown. Green arrows represent downregulation while red arrows represent upregulation in sgKDM4C cells as observed in our current study. Asterisks *, ** and *** represent statistically significantly different between the two groups being compared with *p* < .05, *p* < .01 and *p* < .001, respectively

## CONFLICT OF INTEREST

The authors declare no potential conflicts of interest.

## Supporting information

Supporting information

Supporting information

Supporting information

Supporting information

Supporting information

Supporting information

Supporting information

Supporting information

Supporting information

Supporting information

Supporting information

Supporting information

Supporting information

Supporting information
