# Supplementary figures and images for: Inhibition of KDM4C/c‐Myc/LDHA signalling axis suppresses prostate cancer metastasis via interference of glycolytic metabolism
Source: Clin Transl Med. 2022 Mar 28;12(3):e764. doi: 10.1002/ctm2.764 (PMC8958350; doi:10.1002/ctm2.764)

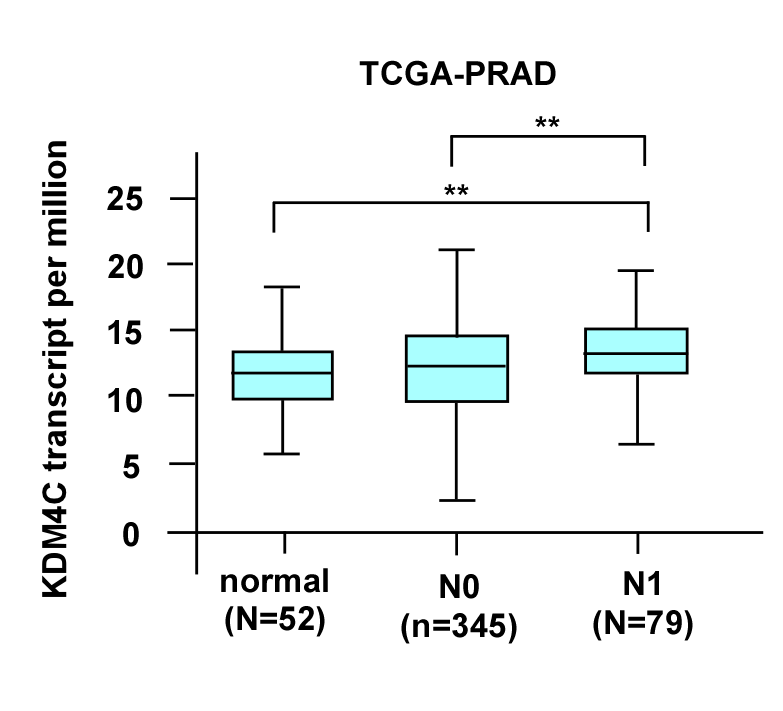

Supplement: Supplementary file 1 — Supporting information [file CTM2-12-e764-s012.tif]

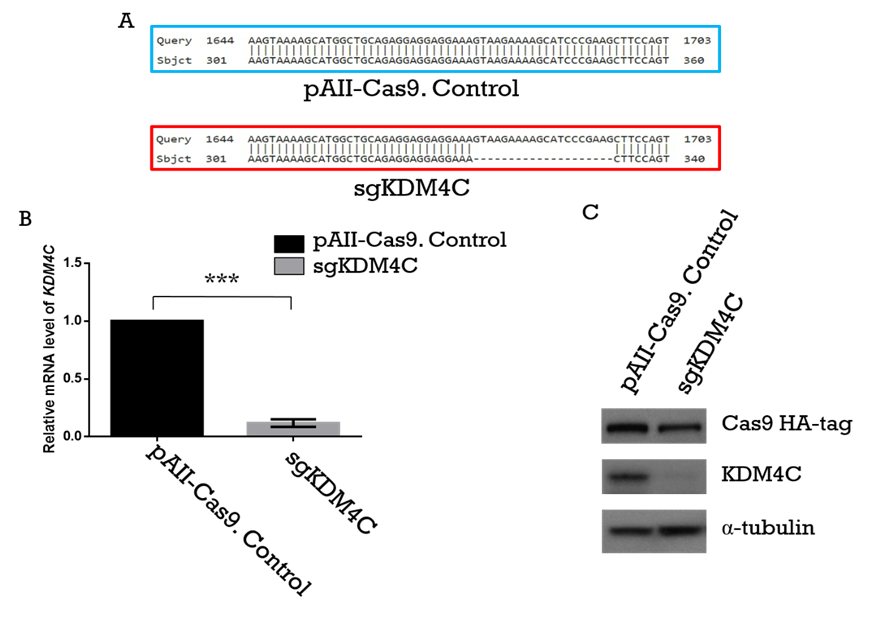

Supplement: Supplementary file 2 — Supporting information [file CTM2-12-e764-s010.tif]

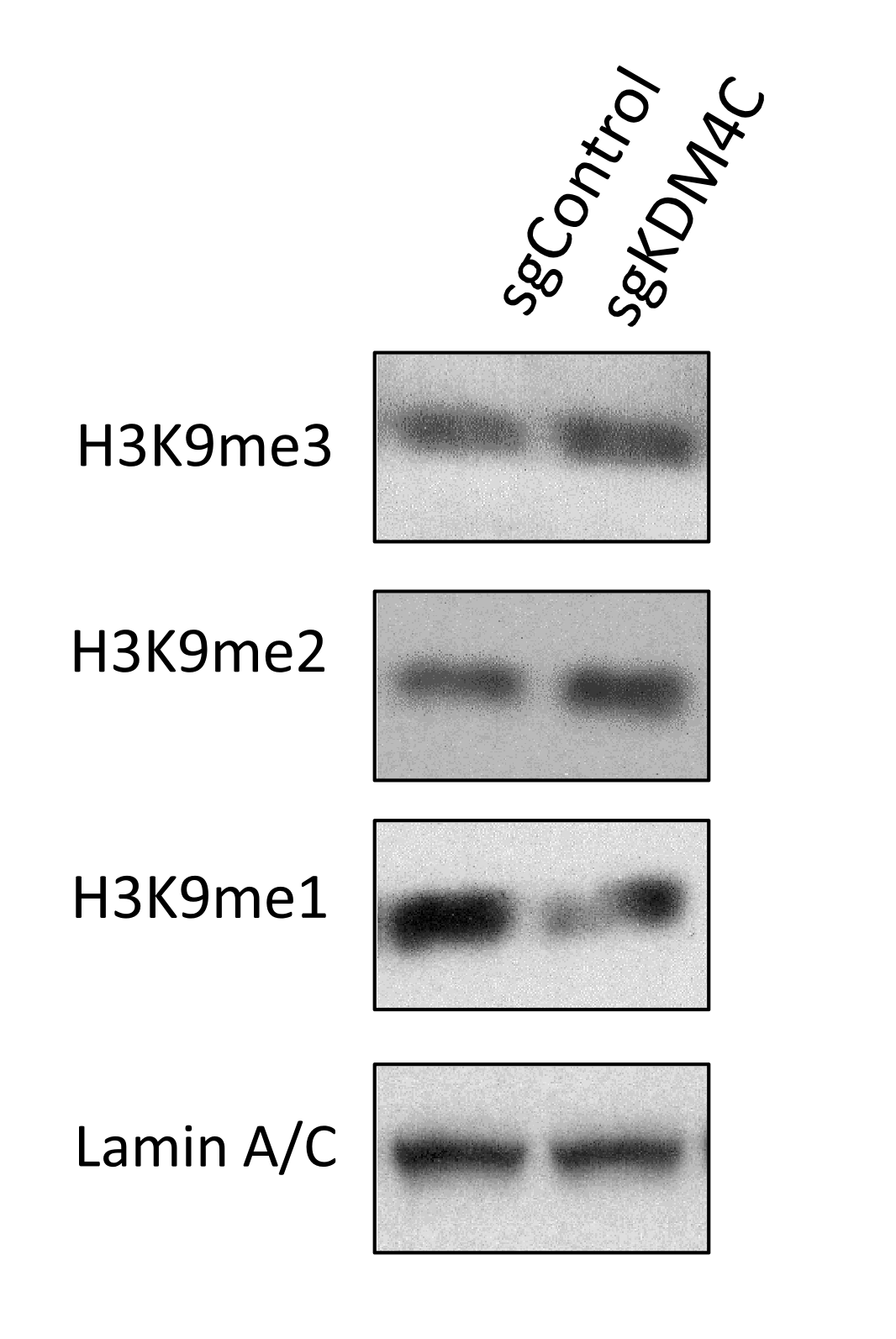

Supplement: Supplementary file 3 — Supporting information [file CTM2-12-e764-s003.tif]

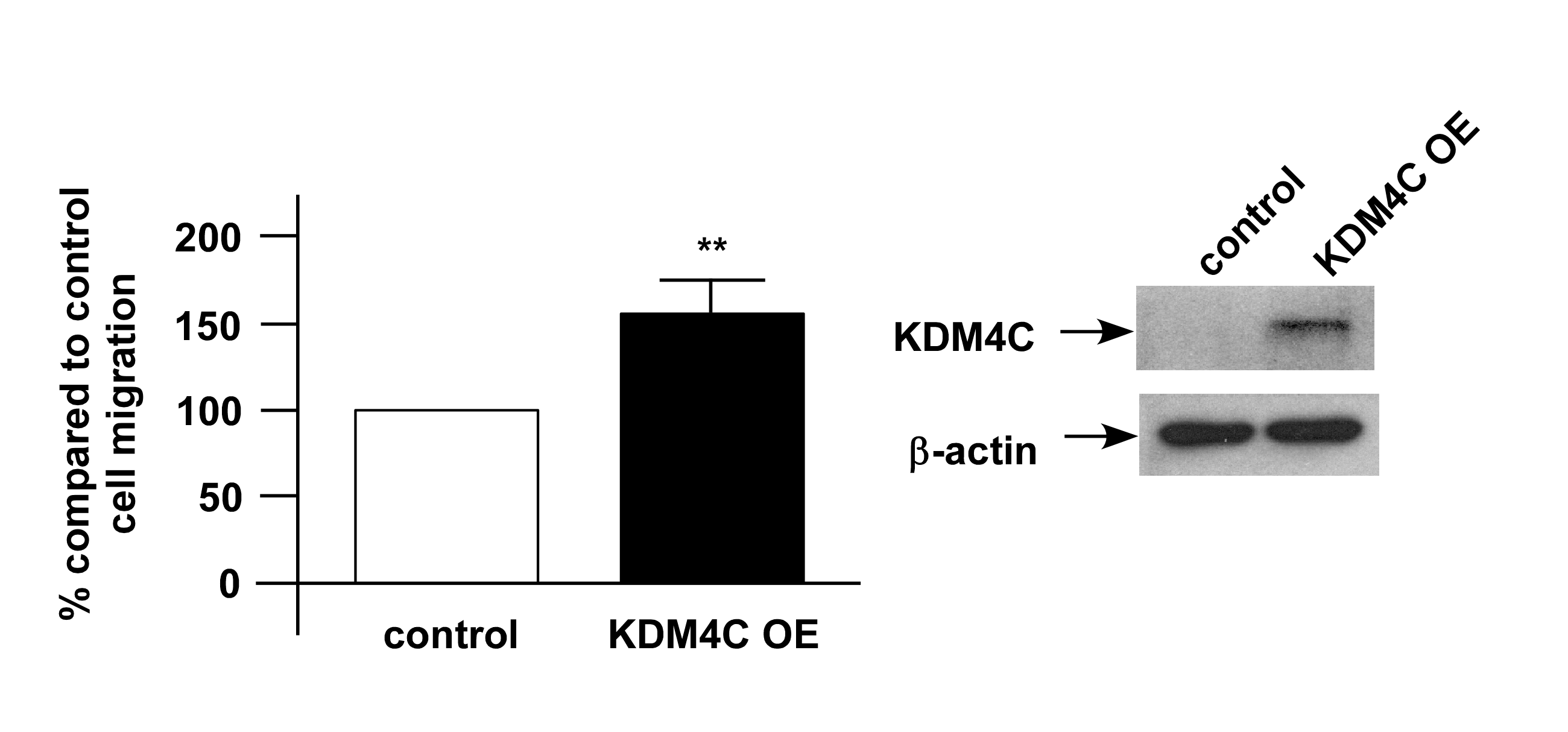

Supplement: Supplementary file 4 — Supporting information [file CTM2-12-e764-s005.tif]

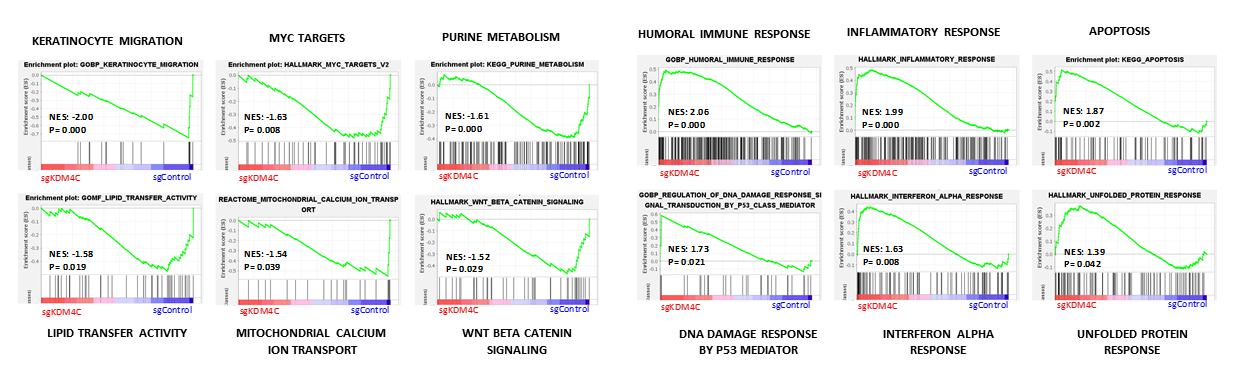

Supplement: Supplementary file 5 — Supporting information [file CTM2-12-e764-s002.tif]

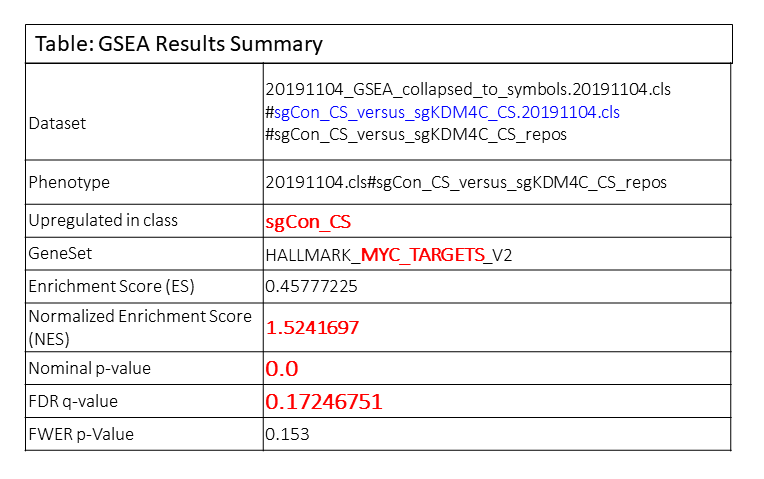

Supplement: Supplementary file 6 — Supporting information [file CTM2-12-e764-s001.tif]

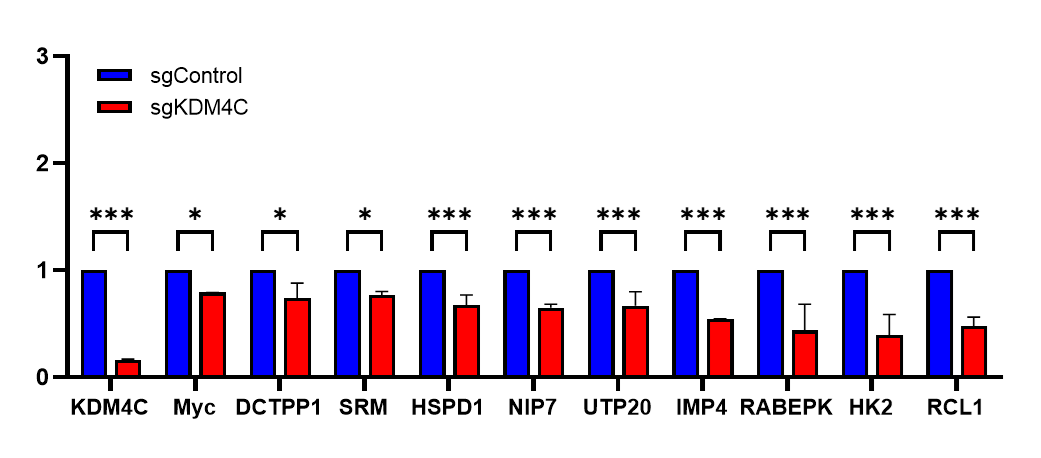

Supplement: Supplementary file 7 — Supporting information [file CTM2-12-e764-s007.tif]

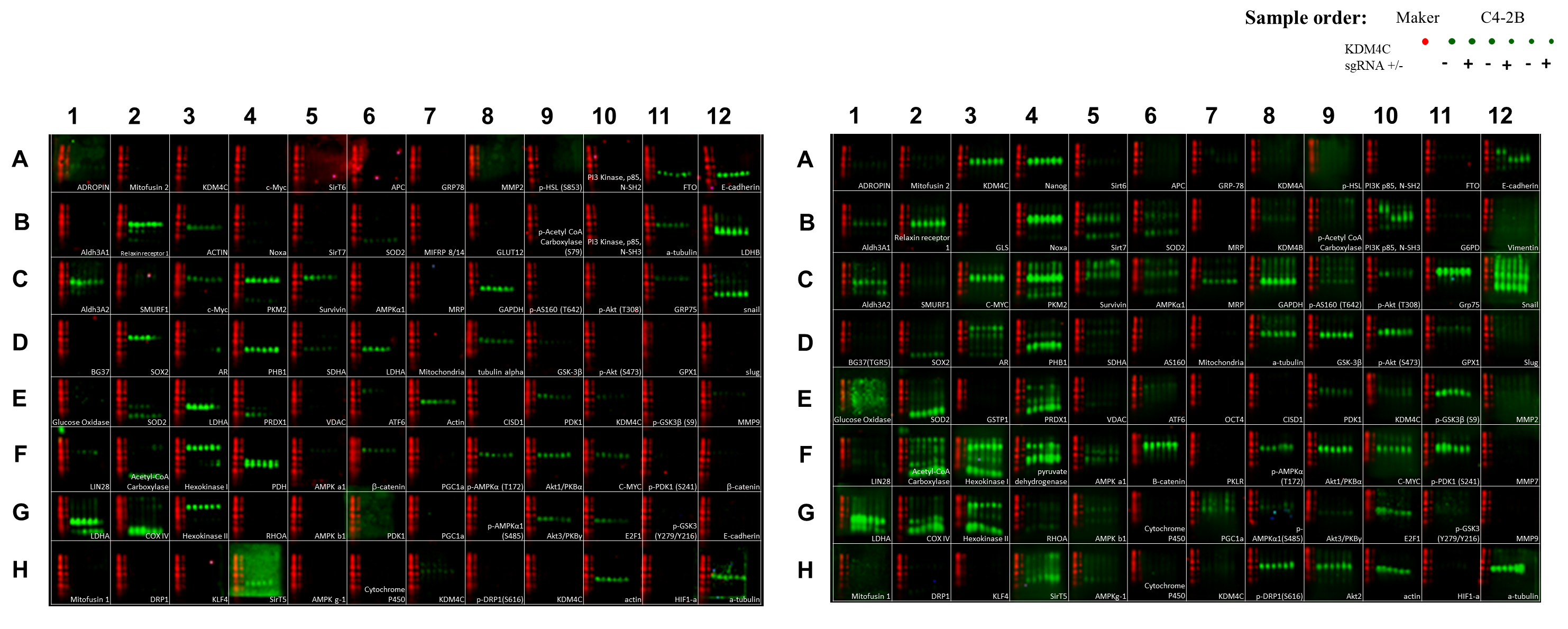

Supplement: Supplementary file 8 — Supporting information [file CTM2-12-e764-s004.tif]

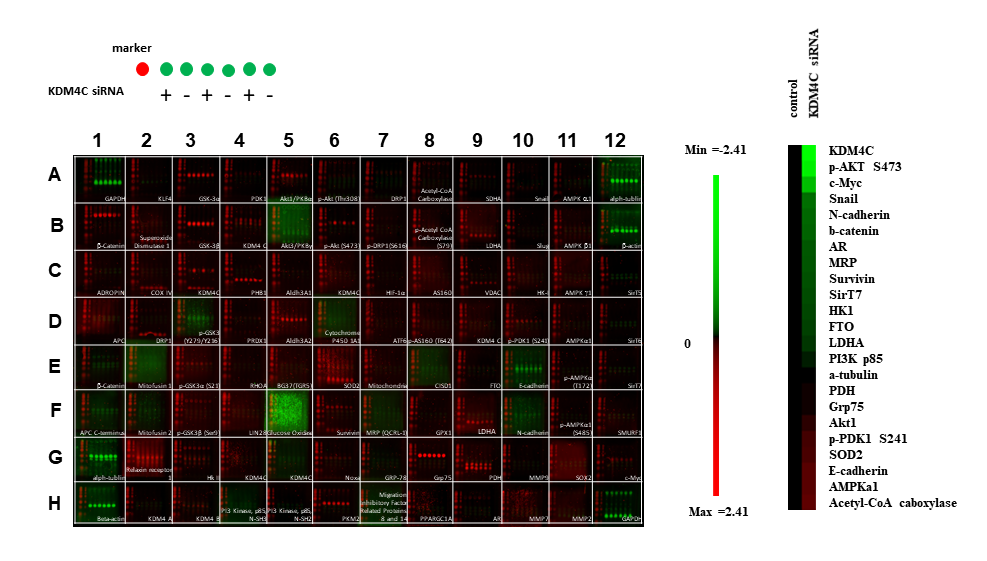

Supplement: Supplementary file 9 — Supporting information [file CTM2-12-e764-s011.tif]

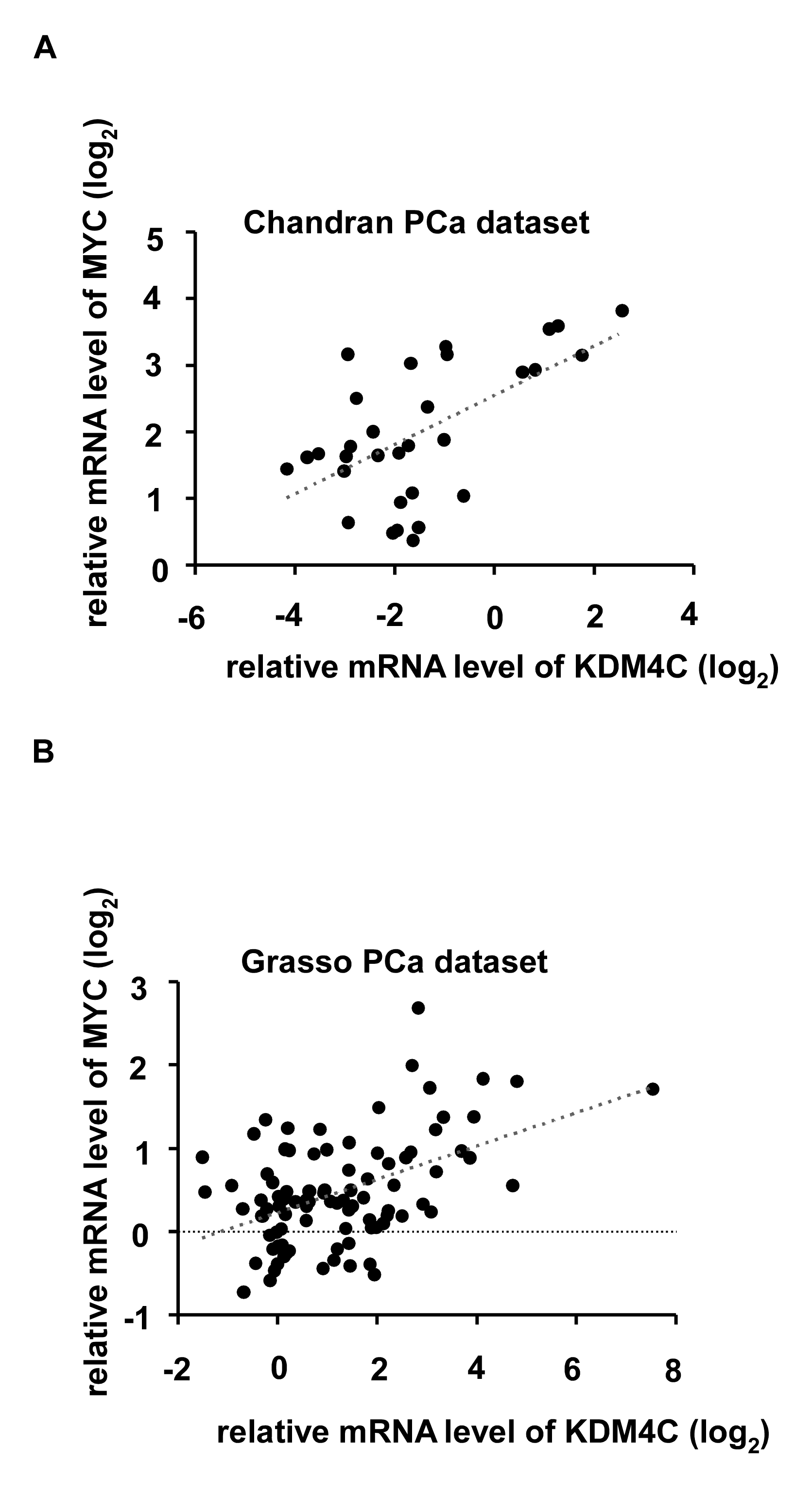

Supplement: Supplementary file 10 — Supporting information [file CTM2-12-e764-s013.tif]

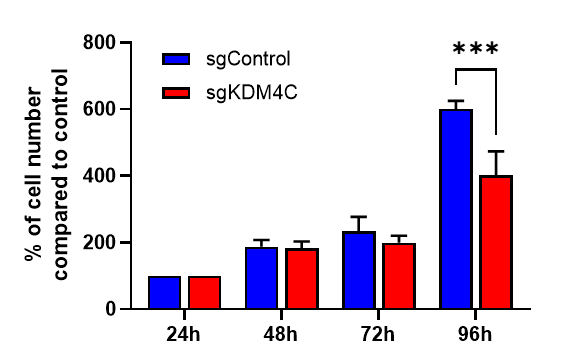

Supplement: Supplementary file 11 — Supporting information [file CTM2-12-e764-s006.tif]

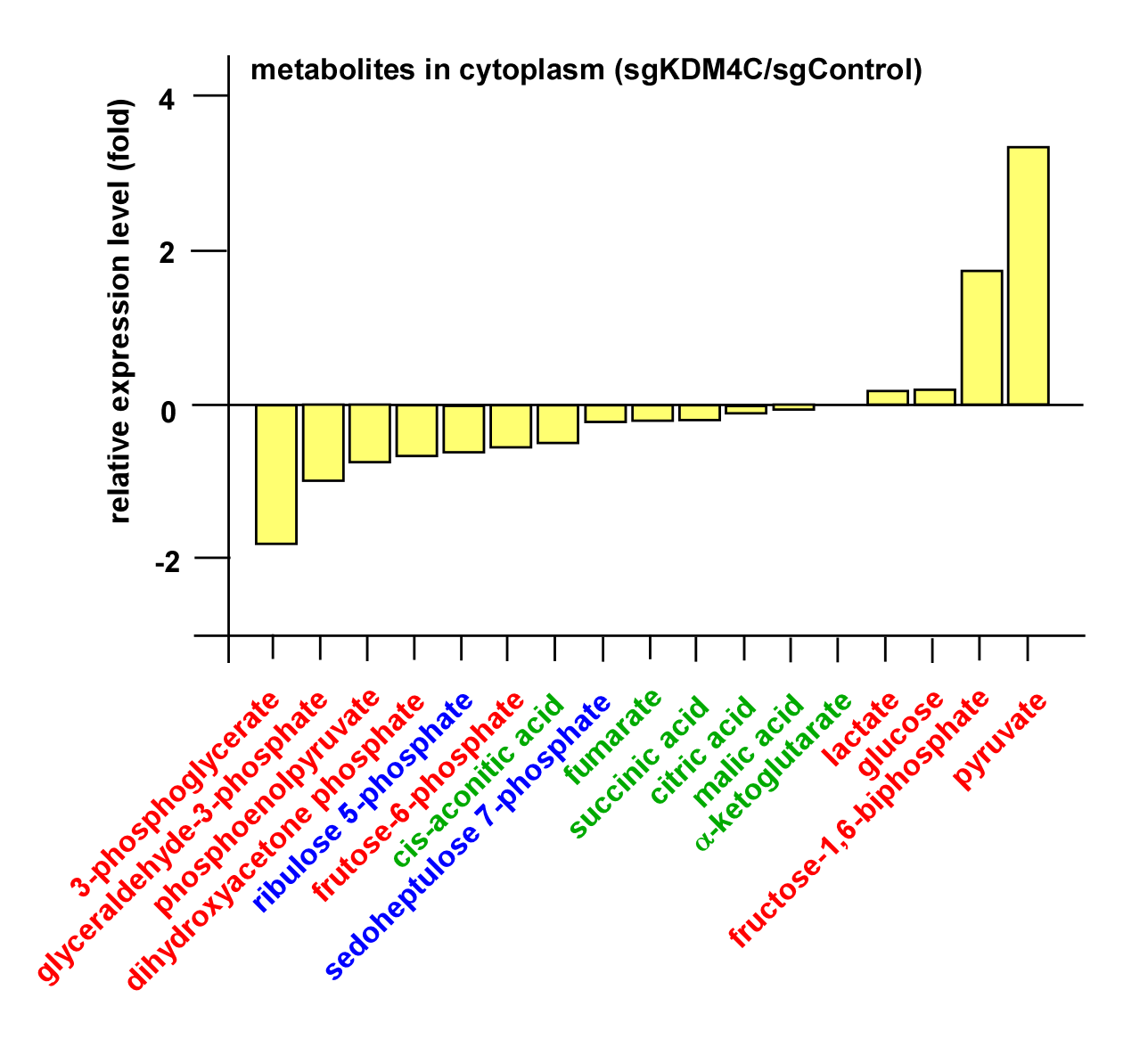

Supplement: Supplementary file 12 — Supporting information [file CTM2-12-e764-s009.tif]
