# Supplementary material for: Inhibition of KDM4C/c‐Myc/LDHA signalling axis suppresses prostate cancer metastasis via interference of glycolytic metabolism
Source: Clin Transl Med. 2022 Mar 28;12(3):e764. doi: 10.1002/ctm2.764 (PMC8958350; doi:10.1002/ctm2.764)
Supplement: Supplementary file 13 — Supporting information [file CTM2-12-e764-s008.docx]

| **Antibody name** | **Company** | **Catalog** |
| --- | --- | --- |
| **Acetyl-CoA Carboxylase** | Cell-signaling | #3662 |
| **ACO1** | Proteintech | 12406-1-AP |
| **ADROPIN** | abcam | ab122800 |
| **Akt1/PKBα** | millipore | 05-796 |
| **Akt2** | Novus | NBP-26594 |
| **Akt3/PKBγ** | millipore | 05-780 |
| **Aldh3A1** | abcam | ab129022 |
| **Aldh3A2** | abcam | ab113111 |
| **AMPKα 1** | Epitomics | 1596-1 (ab32047) |
| **AMPKβ 1** | Epitomics | Jan-89 |
| **AMPKγ 1** | Epitomics | 1592-1 |
| **AMPKα1** | millipore | 04-323 |
| **APC** | Epitomics | 1701-1 (ab40778) |
| **AR** | abcam | ab108341 |
| **AS160** | millipore | 07-741 |
| **ATF6** | millipore | 09-069 |
| **BG37** | abcam | ab72608 |
| **CISD1** | OriGene | TA500905 |
| **c-Myc** | abcam | ab32072 |
| **COX IV** | Cell-signaling | #4850 |
| **Cytochrome P450** | millipore | MAB10111 |
| **Cytochrome P450** | millipore | MABS19 |
| **DRP1** | Cell-signaling | #8570 |
| **E2F1** | millipore | 05-379 |
| **E-cadherin** | Cell-signaling | #4065 |
| **FTO** | Cell-signaling | #14386 |
| **G6PD** | Cell-signaling | #12263 |
| **GAPDH** | Novus | NB300-322 |
| **GGT1** | Novus | H00002678 |
| **GLS** | abcam | ab156876 |
| **Glucose Oxidase** | abcam | ab181638 |
| **GLUT12** | abcam | ab100993 |
| **GPX1** | Cell-signaling | #3206s |
| **Grp75** | Cell-signaling | #3593 |
| **GRP-78** | millipore | MABS475 |
| **GSK-3β** | Cell-signaling | #9315 |
| **GSTP1** | Cell-signaling | #3369s |
| **H3K9me3** | GeneTex | GTX121677 |
| **Hexokinase I** | Cell-signaling | #2024 |
| **Hexokinase II** | Cell-signaling | #2867 |
| **HIF-α** | abcam | ab51608 |
| **KDM4A** | Cell-signaling | #5328 |
| **KDM4B** | Bethyl | A301-478A |
| **KDM4C** | Senta Cruz | sc-515767 |
| **KLF4** | Cell-signaling | #12173 |
| **LDHA** | abcam | ab52488 |
| **LDHB** | Epitomics | #2090-S |
| **LIN28** | abcam | ab46020 |
| **MDH1** | Proteintech | 15904-1-AP |
| **Migration Inhibitory Factor Related Proteins** | millipore | MAB1789 |
| **Mitochondria** | millipore | AB3598 |
| **Mitofusin 1** | abcam | ab57602 |
| **Mitofusin 2** | abcam | ab56889 |
| **MMP2** | abcam | ab37150 |
| **MMP7** | millipore | MAB3322 |
| **MMP9** | abcam | ab76003 |
| **MRP (QCRL-1)** | millipore | 475720-500UL |
| **Nanog** | Cell-signaling | #3580 |
| **Noxa** | Cell-signaling | #14766 |
| **Oct 4** | millipore | MAB4401 |
| **PDHA** | Proteintech | 18068-1-AP |
| **p-Acetyl CoA Carboxylase (S79)** | millipore | 07-303 |
| **p-Akt (S473)** | Cell-signaling | #9271 |
| **p-Akt (T308)** | Cell-signaling | #9275 |
| **p-AMPKα (T172)** | Cell-signaling | #4188 |
| **p-AMPKα1 (S485)** | Cell-signaling | #4184 |
| **p-AS160 (T642)** | millipore | 07-802 |
| **PDH** | Cell-signaling | #3205 |
| **PDK1** | Cell-signaling | #5662 |
| **p-DRP1(S616)** | Cell-signaling | #3455 |
| **PGC-1α** | millipore | AB3242 |
| **p-GSK3 (Y279/Y216)** | millipore | 05-413 |
| **p-GSK3β (Ser9)** | Cell-signaling | #9323 |
| **PHB1** | Cell-signaling | #2426 |
| **p-HSL (S853)** | Epitomics | Jan-38 |
| **PI3 Kinase** | millipore | 05-217 |
| **PI3 Kinase** | millipore | 05-212 |
| **PKLR** | millipore | MABS148 |
| **PKM2** | Cell-signaling | #4053 |
| **p-PDK1 (S241)** | Cell-signaling | #3438 |
| **PRDX1** | Cell-signaling | #8499 |
| **Relaxin receptor 1** | abcam | ab72914 |
| **RHOA** | Cell-signaling | #2117 |
| **SDHA** | Cell-signaling | #11998 |
| **Sirt5** | Cell-signaling | #8782 |
| **Sirt6** | Cell-signaling | #8771 |
| **Sirt7** | Cell-signaling | #5360 |
| **Slug** | Novus | 9585S |
| **SMURF1** | abcam | ab38866 |
| **Snail** | abcam | ab43519 |
| **SOD2** | Genetex | GTX116093 |
| **SOX2** | abcam | ab92494 |
| **Survivin** | Cell-signaling | #2808 |
| **TALDO1** | Proteintech | 12376-1-AP |
| **VDAC** | Cell-signaling | #4661 |
| **Vimentin** | Cell-signaling | 5741S |
| **α-tubulin** | Novus | NB100-690 |
| **β-Actin** | Novus | NB600-501 |
| **β-catenin** | Cell-signaling | #9582 |
