# Supplementary material for: Inhibition of KDM4C/c‐Myc/LDHA signalling axis suppresses prostate cancer metastasis via interference of glycolytic metabolism
Source: Clin Transl Med. 2022 Mar 28;12(3):e764. doi: 10.1002/ctm2.764 (PMC8958350; doi:10.1002/ctm2.764)
Supplement: Supplementary file 14 — Supporting information [file CTM2-12-e764-s014.docx]

Supplemental Table 1

| Name | Forward sequence | Reverse sequence |
| --- | --- | --- |
| ALDOA | ATGCCCTACCAATATCCAGCA | GCTCCCAGTGGACTCATCTG |
| ENO1 | GTGTGGCTCTAACCCTCTGG | TCTGTGACGTTCAGTTTCTTGC |
| GAPDH | AATCCCATCACCATCTTCCAG | CCTTCTCCATGGTGGTGAAGAC |
| GLUT1 | GATTGGCTCCTTCTCTGTGG | TCAAAGGACTTGCCCAGTTT |
| GPI-1 | CAAGGACCGCTTCAACCACTT | CCAGGATGGGTGTGTTTGACC |
| HK2 | GATTGTCCGTAACATTCTCATCGA | TGTCTTGAGCCGCTCTGAGAT |
| LDHA | AGCTGTTCCACTTAAGGCCC | TGGAACCAAAAGGAATCGGGA |
| PDK4 | GAGGATTACTGACCGCCTCTTTAG | TTCCGGGAATTGTCCATCAC |
| PFK | AGATGCGCACCAGCATCAACG | GAACCCGGCACATTGTTGGA |
| PKM2 | GAACTTCTCTCATGGAACTCAT | GATCTCAGGTCCTTTAGTGTCTA |
| PGAM2 | AGAAGCACCCCTACTACAACTC | TCTGGGGAACAATCTCCTCGT |
| PGK | GAACAAGGTTAAAGCCGAGCC | GTGGCAGATTGACTCCTACCA |
| TALDO1 | TCGGTCTTGCTATGTCGAGC | TGTACTCGTCGATGGCGTG |
| ACO | GATATGGGCGCTTACCATTTTCG | TGTGCTGCGTGACATTCCAA |
| CS | GGTGGCATGAGAGGCATGAA | TAGCCTTGGGTAGCAGTTTCT |
| FH | GTGCTGTATTGTCAGGGGAAGC | TGGGATTGGCATTCTCTCCGTC |
| IDH1 | AGAAGCATAATGTTGGCGTCA | CGTATGGTGCCATTTGGTGATT |
| IDH2 | GAAGGTGTGCGTGGAGAC | CCGTGGTGTTCAGGAAGT |
| KGDH | TTGGCTGGAAAACCCCAAAAG | TGTGCTTCTACCAGGGACTGT |
| MDH1 | TTTGGATCACAACCGAGCTAAAG | ACATCTGGATACTGAGTCGAGG |
| PDHA1 | GGGACGTCTGTTGAGAGAGC | TGTGTCCATGGTAGCGGTAA |
| PDHB1 | GGCCACAGTTTGGAGTAGGA | AGCACCAGTGACACGAACAG |
| SDHA | TGGGAACAAGAGGGCATCTG | CCACCACTGCATCAAATTCATG |
| SUCLG1 | GAGCAACGGCTTCTGTCATTT | TGCTTGACTCGTACCATGTCC |
| CANT1 | CTGGGTGTCCAACTACAACG | ACTCCAGCAGGCAGACTCAT |
| NUDT9 | GGCAAGACTATAAGCCTGTG | ATAATGGGATCTGCAGCGTG |
| ACACA | CTGGCTGCATCCATTATGTCA | TGGTAGACTGCCCGTGTGAA |
| ACYL | TGCTCGATTATGCACTGGAAGT | ATGAACCCCATACTCCTTCCCAG |
| CYP11A1 | GAGGGAGACGGGCACACA | TGACATAAACCGACTCCACGTT |
| DHCR24 | GCCGCTCTCGCTTATCTTCG | GTCTTGCTACCCTGCTCCTT |
| FASN | CGCCGACCAGTATAAACCCAA | TCACCCTCAATGATGTGCACA |
| LSS | GGCAGACGTGGACCTACCT | GAAAAGTGGGCCACCATAATC |
| Mlycd | CCTCATGGTCAACTACCGCTACT | CTTGGAGCCCAGGTAGGAGAT |
| GDH | GGAGATGTCCTGGATCGCTG | GTCCATGGATTCCCCCTTGG |
| GGT1 | CTTCTACAACGGCAGCCTCA | TCAGCTCAGCACGGTAGTTG |
| GLS1 | GACATGGAACAGCGGGACTAT | TGTCCTTGGGGAAAGGGTTT |
| GSR | ACGGCATGATAAGGGGATTCA | AGTTTTCGGCCAGCAGCTAT |
| G6PD | ACAGAGTGAGCCCTTCTTCAA | GGAGGCTGCATCATCGTACT |
| PGD | GTACCCGTCACCCTCATTGG | AGAGTGCCTTCCGAATGTCC |
| PGLS | GAGACAGGCATTCCAAGGGG | GTCACTGATGGGAGCCACAA |
| 18S | aaacggctaccacatccaag | cctccaatggatcctcgtta |
| actin | GTACCACTGGCATCGTGATGGACT | CCGCTCATTGCCAATGGTGAT |
| GAPDH | ACAGTCAGCCGCATCTTCTT | ACGACCAAATCCGTTGACTC |
| CDH1 | AGAAACAGGATGGCTGAAGGT | TCTCCATTGGATCCTCAACTG |
| CDH2 | CCTCCAGAGTTTACTGCCATGAC | GTAGGATCTCCGCCACTGATTC |
| MMP2 | CACTTTCCTGGGCAACAAATA | CTTGCGGTCATCATCGTAGTT |
| SNAl1 | TTTACCTTCCAGCAGCCCTA | GACAGAGTCCCAGATGAGCATT |
| CTNNB1 | CACAAGCAGAGTGCTGAAGGTG | GATTCCTGAGAGTCCAAAGACAG |
| VIM | AGGCAAAGCAGGAGTCCACTGA | ATCTGGCGTTCCAGGGACTCAT |
